# Sucrose-free low-fat diet induces metabolic dysfunction through gut dysbiosis and colonic inflammation in mice

**DOI:** 10.3389/fimmu.2026.1813722

**Published:** 2026-07-21

**Authors:** Nourah Almansour, Shihab Kochumon, Fatema Al-Rashed, Md Zubbair Malik, Noelle Benobaid, Steve Shenouda, Reeby Thomas, Hossein Arefanian, Rasheeba Nizam, Texy Jacob, Ashraf Al Madhoun, Jaakko Tuomilehto, Fahd Al-Mulla, Rasheed Ahmad

**Affiliations:** 1 Immunology & Microbiology Department, Dasman Diabetes Institute, Kuwait City, Kuwait; 2 Translational Research Department, Dasman Diabetes Institute, Kuwait City, Kuwait; 3 Animal and Imaging Core Facilities, Dasman Diabetes Institute, Kuwait City, Kuwait; 4 Department of Public Health, University of Helsinki, Helsinki, Finland

**Keywords:** dysbiosis, gut inflammation, gut microbiota, hepatic inflammation, liver steatosis, low-fat diet, metabolic dysfunction, sucrose restriction

## Abstract

Low-fat diets are widely promoted as health-protective; however, the consequences of removing sucrose within a low-fat dietary framework remain unclear. Here, we investigated the effects of a sucrose-free low-fat diet (SF-LFD) compared with a sucrose-containing low-fat control diet (C-LFD) in mice (n=6/group) over 16 weeks. Despite unchanged body and liver weights, SF-LFD feeding resulted in impaired glucose tolerance, reduced insulin sensitivity, and broad alterations in circulating metabolic hormones, including elevated C-peptide, incretins, ghrelin, and resistin, as well as reduced fasting insulin. 16S rRNA sequencing revealed that SF-LFD markedly disrupted gut microbial diversity and composition, with depletion of short-chain fatty acid–producing commensals, including Lactobacillus murinus and members of the Lachnospiraceae family, and enrichment of taxa associated with inflammatory or stress-adapted states, including Helicobacter ganmani, Odoribacter splanchnicus, and Alistipes species. This dysbiosis was accompanied by pronounced colonic inflammation characterized by crypt architectural disruption, loss of goblet cells, submucosal expansion, increased CD3^+^ T-cell and F4/80^+^ macrophage infiltration, and robust upregulation of inflammatory mediators, including *Il1b, Il6, Ccl2, Rorγt*, and *Tbx21*. SF-LFD feeding induced hepatic microvesicular steatosis, lobular inflammation, recruitment of F4/80^+^ and CD11c^+^ immune cells and increased hepatic expression of *IL1b* and *IL6*. Together, these findings suggest that sucrose elimination from a low-fat diet disrupts gut microbiota, impairs metabolic homeostasis, and promotes gut and liver inflammation, revealing an unrecognized dietary trigger of metabolic dysfunction.

## Graphical Abstract

**Figure:**
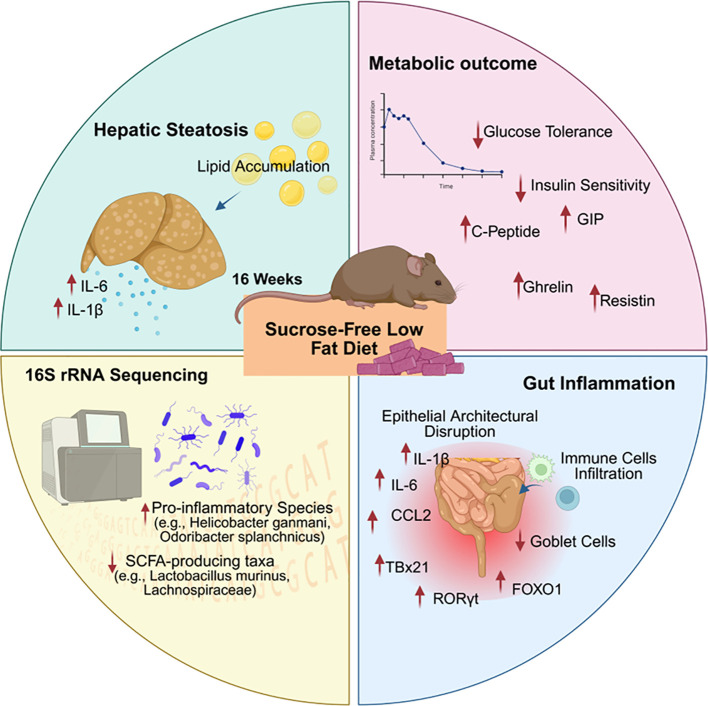

## 1 Introduction

Obesity and metabolic syndrome are overlapping complex disorders, that are both associated with chronic low-grade inflammation (1–3). Obesity is often accompanied with dyslipidemia, hypertension, hypertriglyceridemia, and hyperglycemia. Individuals presenting with at least three of these conditions are considered to have metabolic syndrome (4). The onset of these disorders has been linked to genetic and environmental factors, with one of the major environmental contributors being a high-fat diet (HFD) (5). In fact, HFD-fed mice have been widely used to demonstrate the impact of this diet on progression of obesity and its associated metabolic alterations. Numerous studies have assessed the role of HFD in the synthesis and release of proinflammatory cytokines and adipokines within adipose tissue. The release of these proinflammatory mediators negatively affects peripheral organs, including the liver, bone, and skeletal muscle (6). HFD has also been shown to contribute to hepatic lipid accumulation, which is a hallmark of liver steatosis and non-alcoholic fatty liver disease (NAFLD) (7). Consequently, medical guidelines often recommend a low-fat diet (LFD) as a strategy for achieving and maintaining metabolic homeostasis. Research has shown that an LFD is more effective in reversing and improving HFD-induced insulin resistance in C57BL/6J mice compared with energy restriction alone (8). Although the metabolic consequences of HFD have been extensively studied, there remains limited understanding of the direct role of sucrose in the regulation of metabolic health.

A diet high in sucrose does not directly induce obesity; however, it can promote adipocyte hypertrophy and trigger glucose intolerance, hyperinsulinemia, hyperlipidemia, hepatic steatosis, and elevated levels of inflammatory cytokines. The detrimental impact of such a diet, even when sucrose iso-calorically replaces starch in animal models, can profoundly affect metabolic health (9). It is widely acknowledged that increased sucrose consumption leads to elevated *de novo* fatty acid synthesis, resulting in hepatic triglyceride accumulation (10). This effect occurs primarily because fructose, a component of the sucrose disaccharide, can directly drive *de novo* lipogenesis (11, 12). Accordingly, in addition to fructose-induced hepatic fatty acid synthesis, the progression of steatosis associated with high sucrose intake likely involves fatty acids originating from extrahepatic sources (13, 14). Beyond its direct impact on the liver, high dietary sucrose intake also induces molecular alterations in adipose tissue, leading to dysregulated lipolysis and increased non-esterified fatty acid (NEFA) levels in blood circulation. This, in turn, is likely to increase hepatic fatty acid uptake and re-esterification, further exacerbating lipid accumulation in the liver.

Despite extensive studies examining the effects of high-sucrose diets, low-carbohydrate diets, and ketogenic diets on overall metabolic health (15–19), there remains a limited understanding of how standard sucrose intake affect the colon and its distinct contributions to metabolic health in mice. The present study aimed to address this knowledge gap by investigating the effects of low-fat in the presence and absence of a standard amount of sucrose on the metabolic health of mice.

## 2 Materials & methods

### 2.1 Animals

Animal experimental procedures were performed in accordance with the National Institutes of Health guidelines for the care and use of laboratory animals and were reviewed and approved by the Dasman Institutional Animal Care and Ethics Committee (Approval No. RA AM 2016-007). C57BL/6 mice were purchased from the Jackson Laboratory, and the animals were bred in the Dasman institutional animal facility and fed ad libitum on a standard chow diet. Mice were housed in a temperature-controlled room (23 °C) and maintained on a 12-hour dark/12-hour light cycle. All experiments were performed using 8–10 week old mice. Mice were randomly divided into 2 groups, 6 mice per group, and their body weight was recorded throughout the study. SF-LFD, [9 Kcal (%) fat from soyabean oil] (D18060402, Research Diets Inc.) and C-LFD with normal sucrose [9 Kcal (%) fat and 260 kcal (%) sucrose], (D21120801, Research Diets Inc.) were used (**Supplementary Table 1**). Body weight and food intake of mice were recorded weekly. After 12–16 weeks of dietary intervention, oral glucose tolerance test (OGTT) and insulin tolerance test (ITT) were performed. Mice were sacrificed at the end of the dietary intervention (16 weeks), and all tissues and organs were collected and flash-frozen in liquid nitrogen. Blood was collected for the isolation of plasma and. plasma was stored at −80 °C until use. Liver tissue was fixed in 10% neutral buffered formalin and preserved by paraffin embedding.

### 2.2 Anesthesia and euthanasia

Terminal anesthesia was induced using a combination of ketamine (100 mg/mL) and xylazine (20 mg/mL). A working solution was prepared by diluting 0.9 mL of ketamine and 0.5 mL of xylazine in 8.6 mL of sterile 0.9% normal saline to a total volume of 10 mL, yielding final concentrations of 9 mg/mL and 1 mg/mL, respectively. Mice received a single intraperitoneal (IP) injection at a weight-adjusted volume of 0.01 mL/g, corresponding to a final dosage of 90 mg/kg ketamine and 10 mg/kg xylazine.

The depth of anesthesia was monitored for 2–3 minutes post-injection and strictly verified by the total loss of the righting reflex and the absence of the pedal withdrawal reflex (toe pinch). Dissection and tissue collection were performed only after a surgical plane of anesthesia was confirmed and the absence of pain sensation was ensured. All procedures were conducted in accordance with the Institutional Animal Care and Ethics Committee guidelines.

### 2.3 Oral glucose tolerance test

To perform the glucose tolerance test at 14 weeks of dietary intervention, mice were fasted for 12 hours. Glucose (1 g/kg body weight) was administered orally, and blood glucose levels were measured at 0, 10, 20, 30, 60, 90, and 120 minutes using a portable glucometer.

### 2.4 Insulin tolerance test

To determine insulin tolerance at 12 weeks of dietary intervention, mice were fasted for 4 hours, and insulin (0.8 U/kg body weight) was administered intraperitoneally. Blood glucose levels were determined at 0, 15, 30, 45, 60, 75, 90, 105, and 120 minutes using a portable glucometer.

### 2.5 Plasma measurements

Plasma metabolic hormone levels, including insulin, c-peptide, glucagon, amylin, leptin, PYY, PP, ghrelin, GLP-1, GIP, and resistin, were measured using a MILLIPLEX kit (MILLIPLEX kit MAP mouse Metabolic Magnetic Bead Panel, Millipore) according to the manufacturer’s instructions.

### 2.6 Histological analysis

Liver tissue samples mounted on slides were processed for Hematoxylin-Eosin (H&E) staining and for Oil Red O staining for fat content using standard protocols, as previously described (20, 21) (Methods; **Supplementary File**).

### 2.7 Gene expression analysis by qRT-PCR

Total RNA was extracted from liver tissue using the RNeasy Mini kit (Qiagen, Hilden, Germany) (22). 1 μg of total RNA was reverse-transcribed into cDNA using the High-Capacity cDNA Reverse Transcription Kit (Applied Biosystems, Foster City, USA) (23). Quantitative real-time PCR was performed in a QuantStudio™ 5 Real-Time PCR System using TaqMan master mix reagents and gene-specific TaqMan assays (Applied Biosystems, Foster City, USA). Each reaction was performed in triplicate using standard reaction conditions. Target gene cycle threshold (Ct) values were normalized against GAPDH Ct values, and gene expression level relative to control was calculated using the 2−ΔΔCT method (24). The gene-specific primers are listed in **Supplementary Table 2**.

### 2.8 Microbiome sequencing

Genomic DNA was extracted from mouse fecal samples using the QIAamp DNA Fast Stool Mini Kit (Qiagen, Germany), according to the manufacturer’s instructions. DNA concentration was measured with a Qubit fluorometer (Thermo Fisher Scientific, USA). Bacterial 16S rRNA genes (V3–V4 regions) were amplified from microbial DNA (5 ng/μL) using region-specific primers with adapter overhangs (Forward primer: TCGTCGGCAGCGTCAGATGTGTATAAGAGACAGCCTACGGGNGGCWGCAG Reverse primer: GTCTCGTGGGCTCGGAGATGTGTATAAGAGACAGGACTACHVGGGTATCTAATCC) PCR amplification was done using the KAPA HiFi HotStart ReadyMix PCR Kit (Roche Diagnostics). PCR products were assessed using a Bioanalyzer (Agilent Technologies) using a High Sensitivity DNA chip, purified using AMPure XP beads, and indexed with the Nextera XT Index Kit (Illumina Inc.). Libraries were subsequently purified, normalized, combined (up to 24 samples), and sequenced using paired end reads on the Illumina MiSeq platform.

### 2.9 Microbiome data processing and diversity analysis

Raw 16S rRNA gene sequencing data were processed using the QIIME2 (version: 2025.7) pipeline (25) for quality control, feature table construction, taxonomic assignment, and diversity analysis. Demultiplexed reads were denoised, trimmed, and filtered using the DADA2 plugin to infer high-resolution amplicon sequence variants (ASVs) and remove chimeric sequences. Taxonomic classification was performed using a Naïve Bayes classifier trained on the SILVA 138 reference database (26), trimmed to the appropriate 16S rRNA V3–V4 amplicon region. All 12 fecal samples (n = 6 per group, total n = 12) were included in each diversity analysis. Samples were rarefied to a sequencing depth of 16676 average reads per sample prior to alpha diversity calculation to account for uneven library sizes.

Within-sample (alpha) diversity metrics, including ACE, Chao1, Shannon, Fisher, observed species, and Simpson indices, were calculated to assess microbial richness and evenness within each group. Between group differences in alpha diversity indices were compared using two tailed student’s t-tests (n=6 per group). Because six differences in each alpha diversity indices were compared simultaneously, constituting a multiple-testing scenario, raw p-values were adjusted using the Benjamini–Hochberg (BH) false discovery rate (FDR) correction method. BH-adjusted p-values (q-values) < 0.05 were considered statistically significant for all alpha diversity comparisons.

Between-sample (beta) diversity was estimated using Bray–Curtis dissimilarity, Jaccard distance, and Jensen–Shannon divergence and visualized using Principal Coordinates Analysis (PCoA). The statistical significance of between-group community-level separation was formally assessed using permutational multivariate analysis of variance (PERMANOVA) with 999 permutations, applied independently to each of the three distance matrices within MicrobiomeAnalyst. PERMANOVA outputs, including pseudo-F statistics and permutation-based p-values, are reported in the Results.

### 2.10 Statistical analysis

Statistical analyses were performed using GraphPad Prism (version 9.0, GraphPad software, La Jolla, CA, USA) for all metabolic, histological, and gene expression data. Microbiome-specific statistical analyses were conducted in R (version 4.4), and MicrobiomeAnalyst platform (27).

For repeated-measures outcomes, including weekly body weight-trajectories, OGTT, and ITT time-courses, a two-way repeated-measures ANOVA or a mixed-effects model (REML) was employed. The mixed-effects model was specifically utilized for datasets with missing values to ensure the inclusion of all available data points in the trajectory analysis. Šídák’s multiple comparisons test was used for *post-hoc* analysis to identify differences between groups at specific time points. For comparisons of a single parameter between two groups including Area Under the Curve (AUC), mRNA expression, plasma metabolic hormones, organ weights, and histological staining intensity or infiltration data an unpaired two-tailed Student’s t-test was applied (n = 6 per group). As these represent individual, pre-specified hypothesis tests rather than simultaneous multiple comparisons, no FDR adjustment was required or applied to these analyses. All data are presented as mean ± SEM, and a P value < 0.05 was considered statistically significant.

Differential abundance analysis was performed using Linear Discriminant Analysis Effect Size (LEfSe) within MicrobiomeAnalyst (27), with a minimum LDA score threshold of 2.0 applied to identify biologically meaningful taxon-level differences. Because LEfSe simultaneously evaluates differential abundance across hundreds of microbial taxa, constituting a high-dimensional multiple-testing problem, all LEfSe-derived raw p-values were adjusted using the Benjamini–Hochberg (BH) false discovery rate (FDR) correction method. Only taxa satisfying both a BH-adjusted q-value < 0.05 and an LDA score > 2.0 were considered statistically significant and are reported in the Results. Taxonomic bar plots, diversity indices, and LDA score plots were generated using default MicrobiomeAnalyst settings unless otherwise specified.

All data are presented as mean ± SEM. A p-value < 0.05 was considered statistically significant for all metabolic, histological, and gene expression analyses. For microbiome analyses involving multiple simultaneous comparisons, a Benjamini–Hochberg FDR-adjusted q-value < 0.05 was applied as the significance threshold.

## 3 Results

### 3.1 Sucrose-free LFD impairs glucose homeostasis and alters metabolic hormone profiles

High-fat diets containing sucrose are well known to promote metabolic dysfunction; however, the metabolic impact of a sucrose-free low-fat diet (SF-LFD) remains unclear. To address this, we compared the effects of an SF-LFD with a sucrose-containing low-fat diet (C-LFD) on metabolic parameters. To test this hypothesis, mice were fed either C-LFD or SF-LFD for 16 weeks.

**Figure 1:**
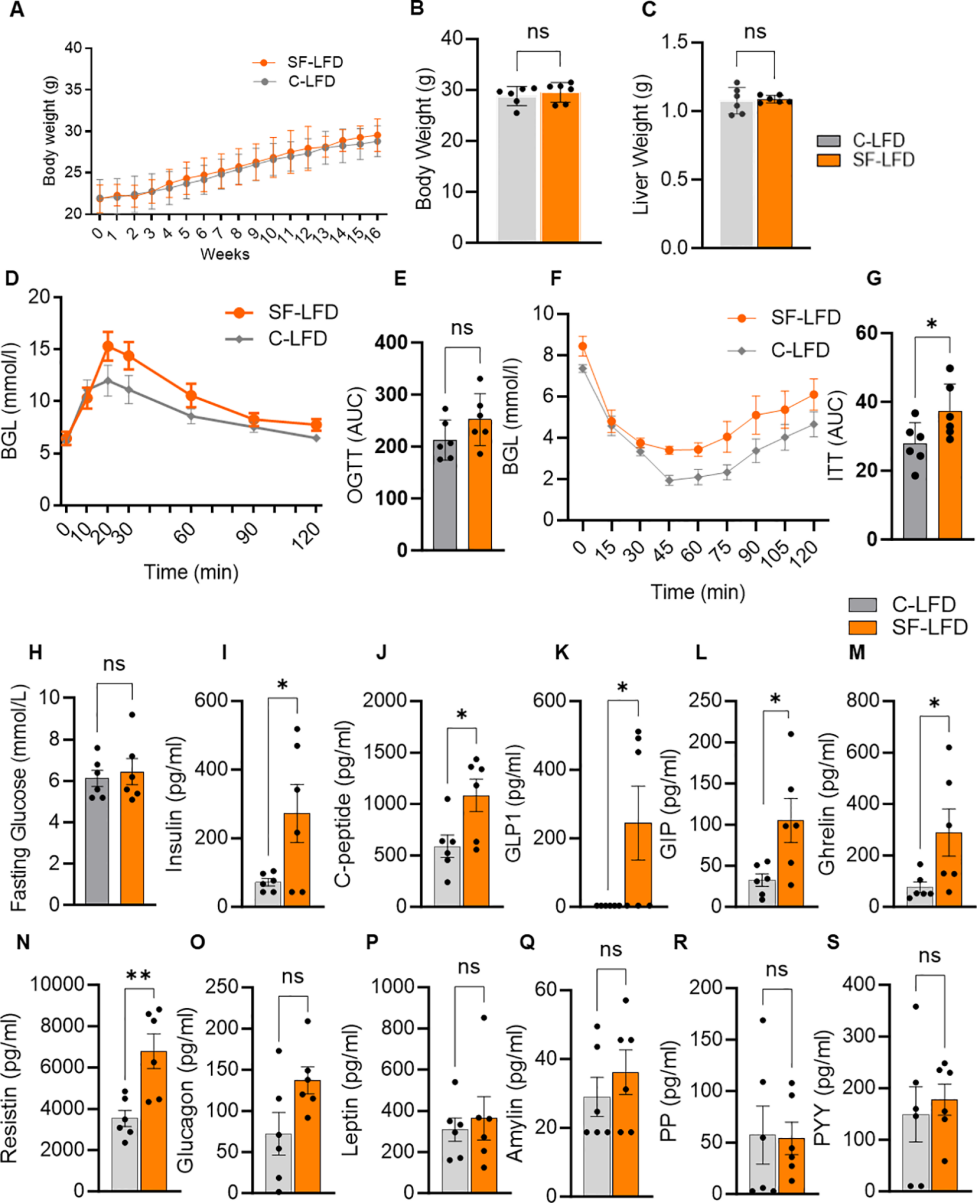
Sucrose-free low-fat diet (SF-LFD) impairs glucose and insulin tolerance in mice. Eight- to ten-week-old male C57BL/6 mice (n = 6 per group) were fed a sucrose-free low-fat diet (SF-LFD) or a control low-fat diet (C-LFD) for 16 weeks. **(A–B)** Body weight. **(C)** Liver weight. Data are expressed as mean ± SD; ns, not significant. **(D–E)** Oral glucose tolerance test (OGTT) was performed after 14 weeks of diet feeding and corresponding area under the curve (AUC) analysis. **(F–G)** Insulin tolerance test (ITT; i.p.) conducted after 12 weeks of diet feeding and corresponding AUC analysis. **(H–I)** Fasting plasma glucose and insulin levels. **(J–S)** Fasting plasma metabolic markers measured using Milliplex assay kits. Data are presented as mean ± SEM. *P < 0.05, **P<0.001, ns, not significant.

Weekly body weight monitoring followed by mixed-effects analysis revealed a significant effect of time (P < 0.0001), reflecting normal growth in both groups, but no significant effect of dietary treatment (P = 0.7721) or time × treatment interaction (P = 0.9652), indicating that sucrose removal did not alter body-weight trajectory (**Figure 1A**). Final body weight and liver weight were also comparable between groups (**Figures 1B, C**). Likewise, food intake did not differ significantly between the two groups throughout the study (**Supplementary Figure 1**), indicating that the observed metabolic differences were independent of caloric intake.

Despite similar body weight, mice fed the SF-LFD exhibited marked disturbances in glucose regulation. During the oral glucose tolerance test (OGTT), two-way repeated-measures ANOVA showed a significant effect of time (P < 0.0001) and a significant time × diet interaction (P = 0.0047), indicating altered glucose handling in the SF-LFD group (**Figure 1D**). *Post hoc* Šídák analysis identified significantly higher blood glucose levels at 20 minutes (P = 0.0010) and 30 minutes (P = 0.0013) in SF-LFD-fed mice. Consistently, total glucose exposure, assessed by area under the curve (AUC), was increased in the SF-LFD group, confirming impaired glucose tolerance (**Figure 1E**).

Insulin sensitivity was further assessed by insulin tolerance testing (ITT). Mixed-effects analysis demonstrated a significant effect of time (P < 0.0001), consistent with the expected hypoglycemic response (**Figure 1F**). Although the main effect of diet (P = 0.0836) and the interaction term (P = 0.5482) were not statistically significant, mice fed the control diet maintained lower glucose levels during the recovery phase. Importantly, AUC analysis revealed significantly greater glucose excursion in the SF-LFD group compared with controls (P < 0.05), indicating reduced insulin responsiveness (**Figure 1G**).

Fasting blood glucose levels showed a non-significant upward trend in SF-LFD-fed mice (**Figure 1H**), whereas fasting insulin levels were significantly altered (**Figure 1I**), suggesting early disruption of endocrine glucose regulation despite the absence of overt changes in body weight.

To further characterize metabolic dysregulation, circulating hormone profiles were assessed. SF-LFD-fed mice exhibited significantly elevated plasma levels of C-peptide, GLP-1, GIP, ghrelin, and resistin compared with controls (**Figures 1J–N**), reflecting enhanced incretin activation, altered appetite signaling, and adipokine imbalance. Glucagon levels were modestly increased but did not reach statistical significance (**Figure 1O**). No significant differences were observed in leptin, amylin, pancreatic polypeptide (PP), or peptide YY (PYY) between dietary groups (**Figures 1P–S**).

Collectively, these findings demonstrate that removal of sucrose within a low-fat dietary framework impairs glucose tolerance, reduces insulin sensitivity, and disrupts multiple metabolic hormone pathways, indicating a metabolically adverse state likely driven by intestinal and hepatic inflammatory stress.

### 3.2 SF-LFD alters gut microbial diversity, community structure, and key functional taxa

**Figure 2:**
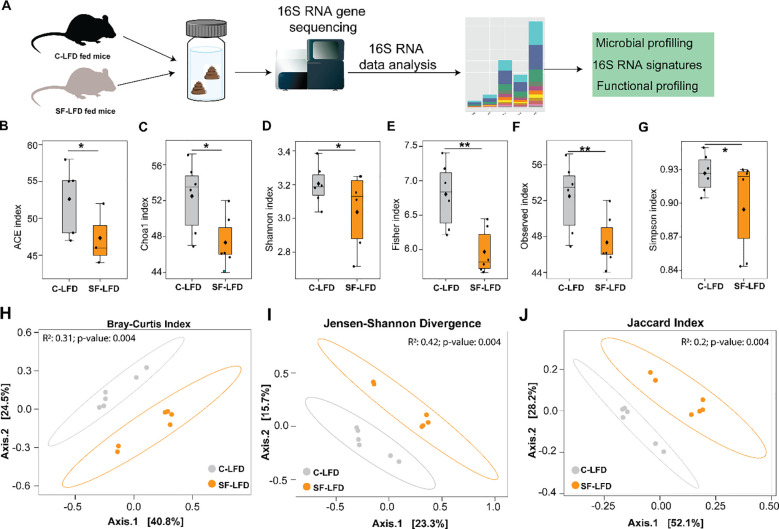
SF-LFD disrupts gut microbiota diversity and community in mice. **(A)** Schematic overview of the experimental workflow, including dietary intervention [control low-fat diet (C-LFD) vs. sucrose-free low-fat diet (SF-LFD)], fecal sample collection, 16S rRNA gene sequencing, and downstream microbial and functional profiling. **(B–G)** Alpha diversity metrics (ACE, Chao1, Shannon, Fisher, Observed species, and Simpson indices) in SF-LFD–fed mice compared with C-LFD–fed controls. Statistical comparisons were performed using unpaired two-tailed Student’s t-tests; p-values were adjusted using the Benjamini–Hochberg false discovery rate (FDR) correction method. Adjusted q-values are indicated on each panel; q < 0.05 was considered statistically significant. **(H–J)** Principal coordinates analysis (PCoA) based on Bray–Curtis dissimilarity, Jensen–Shannon divergence, and Jaccard index reveals distinct clustering by diet, with C-LFD (gray circles) and SF-LFD (orange circles). The statistical significance of between-group community separation was assessed using permutational multivariate analysis of variance (PERMANOVA; 999 permutations). PERMANOVA p-values are displayed on each plot (Bray–Curtis: F = 0.31, p = 0.004; Jensen–Shannon: F = 0.42, p = 0.004; Jaccard: F = 0.20, p = 0.004).Eight- to ten-week-old male C57BL/6 mice (n = 6 per group) were fed a sucrose-free low-fat diet (SF-LFD) or a control low-fat diet (C-LFD) for 16 weeks. Fecal samples were collected at endpoint for 16S rRNA gene sequencing. Data are presented as mean ± SEM (n = 6 per group).

Since the gut microbiota is highly responsive to changes in dietary carbohydrate intake, and given its central role in metabolic regulation, immune function, and intestinal homeostasis, we performed 16S rRNA gene sequencing on fecal samples from mice fed either C-LFD or SF-LFD for 16 weeks (**Figure 2A**).

SF-LFD feeding resulted in a marked reduction in microbial richness and evenness. Alpha diversity metrics such as ACE, Chao1, Shannon, Fischer, Observed and Simpson indices were significantly reduced in microbial diversity in SF-LFD-fed mice compared to C-LFD-fed mice, suggesting a dysbiotic state in the absence of sucrose (**Figures 2B–G**). Beta diversity analyses further confirmed significant community shifts, with distinct separation between the two diet groups (**Figures 2H–J**).

**Figure 3:**
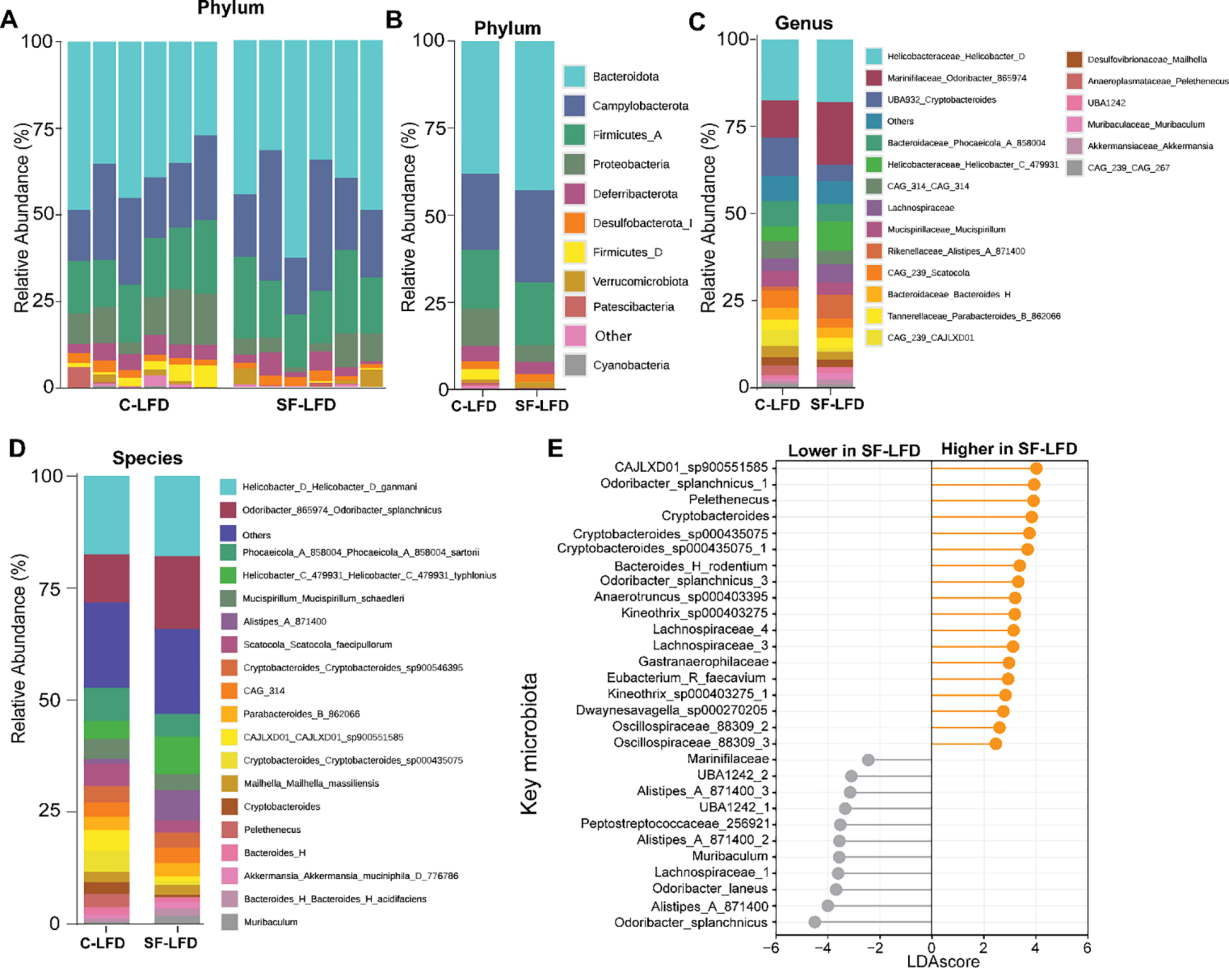
SF-LFD alters gut microbiota composition and enriches distinct pro-inflammatory taxa. **(A)** Relative abundance of bacterial taxa at the phylum level across individual samples. **(B)** Group-averaged relative abundance of bacterial taxa at the phylum level. Summarizing community-level differences between C-LFD and SF-LFD groups **(C)** Genus-level relative abundance illustrating diet-dependent compositional shifts between C-LFD and SF-LFD groups. **(D)** Species-level distributions highlighting taxa differentially represented between C-LFD and SF-LFD groups. **(E)** Linear discriminant analysis effect size (LEfSe) plot identifying abundant between dietary groups. Only taxa meeting both a benjamini-Hochberg FDR-adjusted p- value <0.05 and a minimum LDA score >2.0 are shown. Orange bars denote taxa enriched in the SF-LFD group, gray bars indicate taxa enriched in the C-LFD group. Data are presented as mean ± SEM (n = 6 per group).

At the phylum level, SF-LFD-fed mice exhibited increased relative abundances of Bacteroidota and Campylobacterota and a reduction in Firmicutes compared with C-LFD-fed mice (**Figures 3A, B**), reflecting disruption of major microbial groups involved in energy harvest and metabolic regulation. Genus-level analyses revealed enrichment of Helicobacter, Odoribacter, and Mailhella in the SF-LFD group, whereas Lactobacillus and Ruminococcus were more abundant in C-LFD-fed mice (**Figure 3C**). Species-level profiling further demonstrated increased abundance of Helicobacter ganmani, Odoribacter splanchnicus, and Mailhella massiliensis with SF-LFD feeding, while beneficial taxa such as Lactobacillus murinus and Bacteroides acidifaciens predominated in control mice (**Figure 3D**).

Linear discriminant analysis effect size (LEfSe) analysis identified key discriminatory taxa driving the observed microbial differences between dietary groups (**Figure 3E**). SF-LFD-fed mice were enriched with Odoribacter splanchnicus variants, Cryptobacteroides, Pelotomaculum, Kineothrix, and multiple Alistipes species—taxa frequently associated with intestinal inflammation, bile acid dysregulation, and metabolic stress. Several unclassified but metabolically active taxa (e.g., UBA1142, UBA121) were also enriched, suggesting the emergence of stress-adapted microbial communities under sucrose-deprived conditions. In contrast, the C-LFD group exhibited higher relative abundances of putative protective taxa, including Lactobacillus murinus, Peptostreptococcaceae_2569211, Eubacterium faecivivens, Oscillospiraceae_883039, and Lachnospiraceae_4, which are commonly linked to mucosal integrity and short-chain fatty acid (SCFA) production.

Taken together, these findings demonstrate that removal of sucrose from a low-fat diet profoundly disrupts gut microbial diversity and composition, depletes SCFA-producing and anti-inflammatory commensals, and enriches potentially pathogenic or stress-adapted taxa. These microbiome alterations likely contribute to intestinal inflammation, hepatic steatosis, and metabolic dysfunction observed in SF-LFD-fed mice.

### 3.3 SF-LFD promotes colonic inflammation

Given that SF-LFD feeding induced a dysbiotic microbial profile characterized by depletion of SCFA-producing commensals and enrichment of potentially pro-inflammatory taxa (e.g., Helicobacter ganmani, Odoribacter splanchnicus), we next investigated whether these microbial shifts were associated with structural and immunological changes in the colon. As the colon represents the primary site of interaction among dietary components, microbial metabolites, and the mucosal immune system, we examined histological and inflammatory responses in colonic tissues from mice fed SF-LFD or C-LFD.

**Figure 4:**
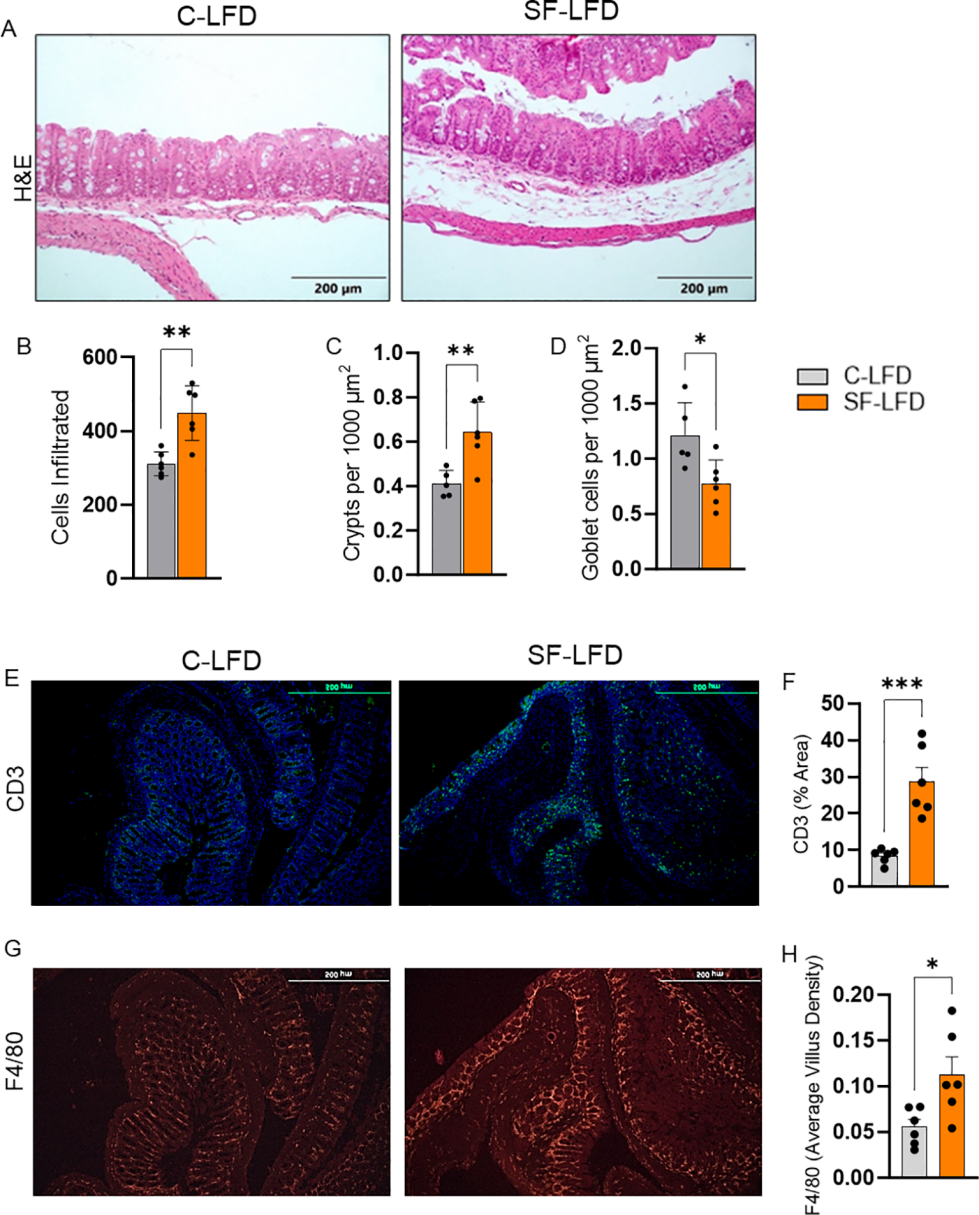
SF-LFD induces histological and immunological alterations in the colon. Eight- to ten-week-old male C57BL/6 mice (n = 6 per group) were fed a sucrose-free low-fat diet (SF-LFD) or control low-fat diet (C-LFD) for 16 weeks. Following dietary intervention, mice were euthanized and colonic tissues were collected. **(A)** Representative hematoxylin and eosin (H&E)–stained sections of distal colon from C-LFD–fed (left) and SF-LFD–fed (right) mice. Scale bars, 200 μm. **(B)** Quantification of infiltrating inflammatory cells. **(C-D)** Histomorphometric analysis was performed using ImageJ software to quantify the number of crypts and goblet cells per 1000 µm² in histological sections from all animals. **(E)** Immunofluorescence staining of CD3^+^ T cells (blue) in colonic sections from C-LFD–fed (left) and SF-LFD–fed (right) mice. **(F)** Quantification of CD3^+^ T cell infiltration. **(G)** Immunofluorescence staining of F4/80^+^ macrophages (red) in colonic sections from C-LFD–fed (left) and SF-LFD–fed (right) mice. **(H)** Quantification of F4/80^+^ macrophage infiltration. Data are presented as mean ± SEM (n = 6 per group). Statistical significance was determined by unpaired two-tailed Student’s *t* test: *P < 0.05, *P < 0.01, **P < 0.001, ***P<0.0001.

Histological evaluation of colonic tissues from C-LFD–fed mice revealed normal mucosal architecture, characterized by an intact epithelium, well-organized and elongated crypts, and abundant goblet cells (**Figures 4A–D**). In contrast, SF-LFD–fed mice displayed pronounced architectural disruptions, including irregular and less densely packed crypts, reduced goblet cell numbers, and expansion of the submucosal layer, suggestive of early inflammatory remodeling (**Figures 4A–D**). These structural abnormalities indicate that sucrose elimination from a low-fat diet compromises colonic epithelial homeostasis and may impair barrier integrity (**Figures 4A–D**). Consistent with this interpretation, colonic tissues from SF-LFD-fed mice showed increased inflammatory cell infiltration.

Immunophenotyping further revealed that SF-LFD markedly altered the colonic immune landscape. SF-LFD–fed mice exhibited significant increases in CD3^+^ T cells (**Figures 4E, F**) and F4/80^+^ macrophages within the lamina propria (**Figures 4G, H**), changes that were not observed in C-LFD mice.

**Figure 5:**
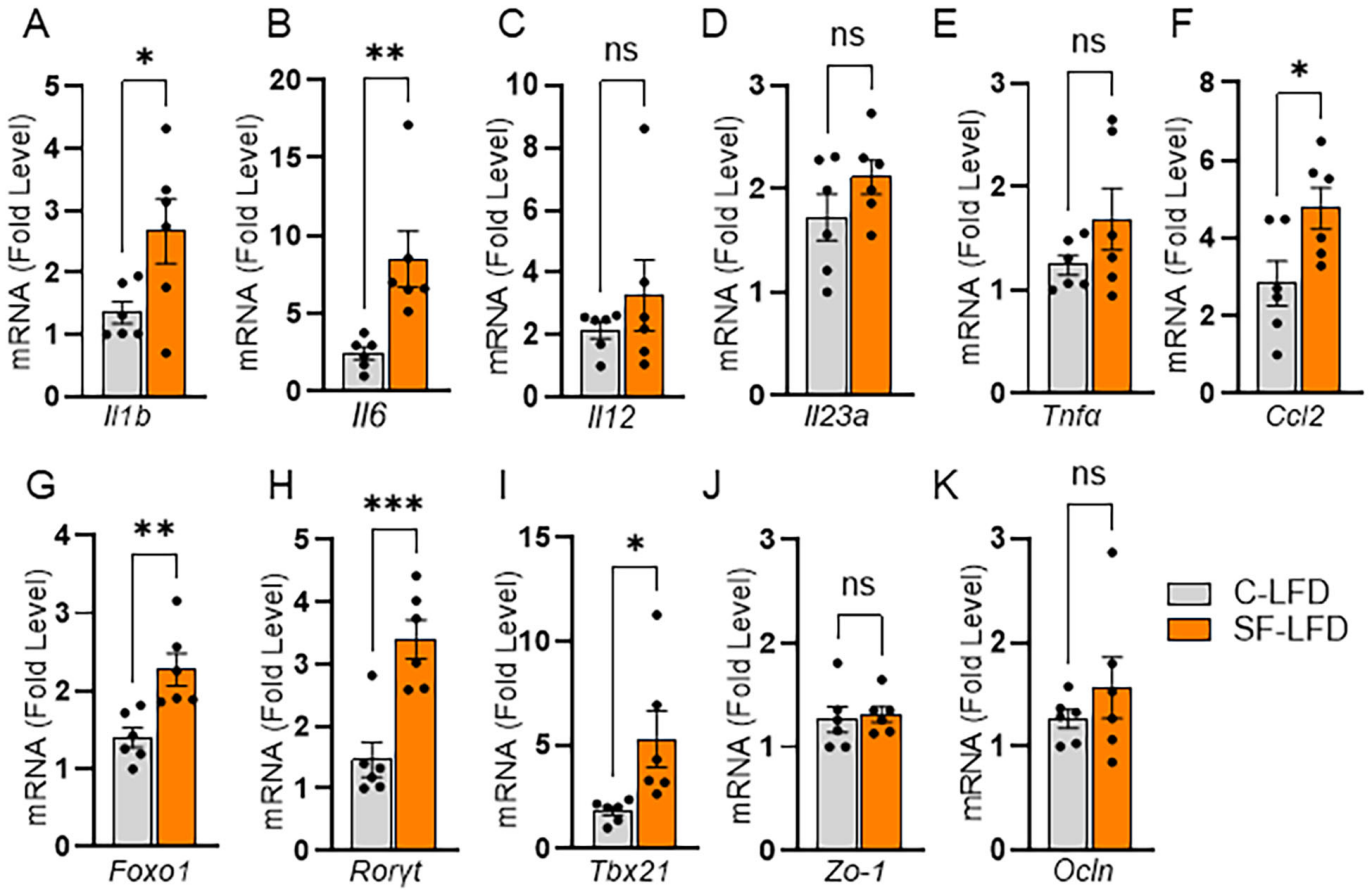
Colonic gene expression in mice fed SF-LFD or C-LFD. Male mice were fed either a sucrose-free low-fat diet (SF-LFD) or a control low-fat diet (C-LFD) for 16 weeks. Colonic tissues were collected, total RNA was isolated, and quantitative RT-PCR **(A–K)** was performed to assess the expression of proinflammatory cytokines (Il1β, Il6, Il12, Il23a, and Tnfα), the chemokine Ccl2, transcription factors (Foxo, Rorγt, and Tbx21), and epithelial permeability markers (Zo-1 and Occludin). Gene expression was normalized to Gapdh and expressed relative to the C-LFD group. Data are presented as mean ± SEM (n = 6). Statistical significance was determined by unpaired two-tailed Student’s t test: *P < 0.05, *P < 0.01, **P < 0.001, ***P<0.0001, ns, not significant.

Colon inflammation in SF-LFD–fed mice was further supported by transcriptional profiling of inflammatory mediators. Consistent with the histological findings, colonic expression of *Il1b, Il6, Ccl2, Foxo1, Rorγt*, and *Tbx21* was significantly elevated in SF-LFD mice compared with C-LFD mice. Although *Il12, Il23a* and *Tnfa* showed upward trends, these changes did not reach statistical significance (**Figures 5A–I**). Expression of epithelial barrier genes *Zo-1* and *Ocln* remained unchanged between the two groups, indicating that SF-LFD–induced inflammation occurred without major alterations in tight junction gene expression (**Figures 5J, K**).

Together, these findings demonstrate that SF-LFD induces a pronounced inflammatory phenotype in the colon, linking diet-induced dysbiosis to mucosal immune activation and epithelial remodeling.

### 3.4 SF-LFD drives hepatic steatosis and inflammation

**Figure 6:**
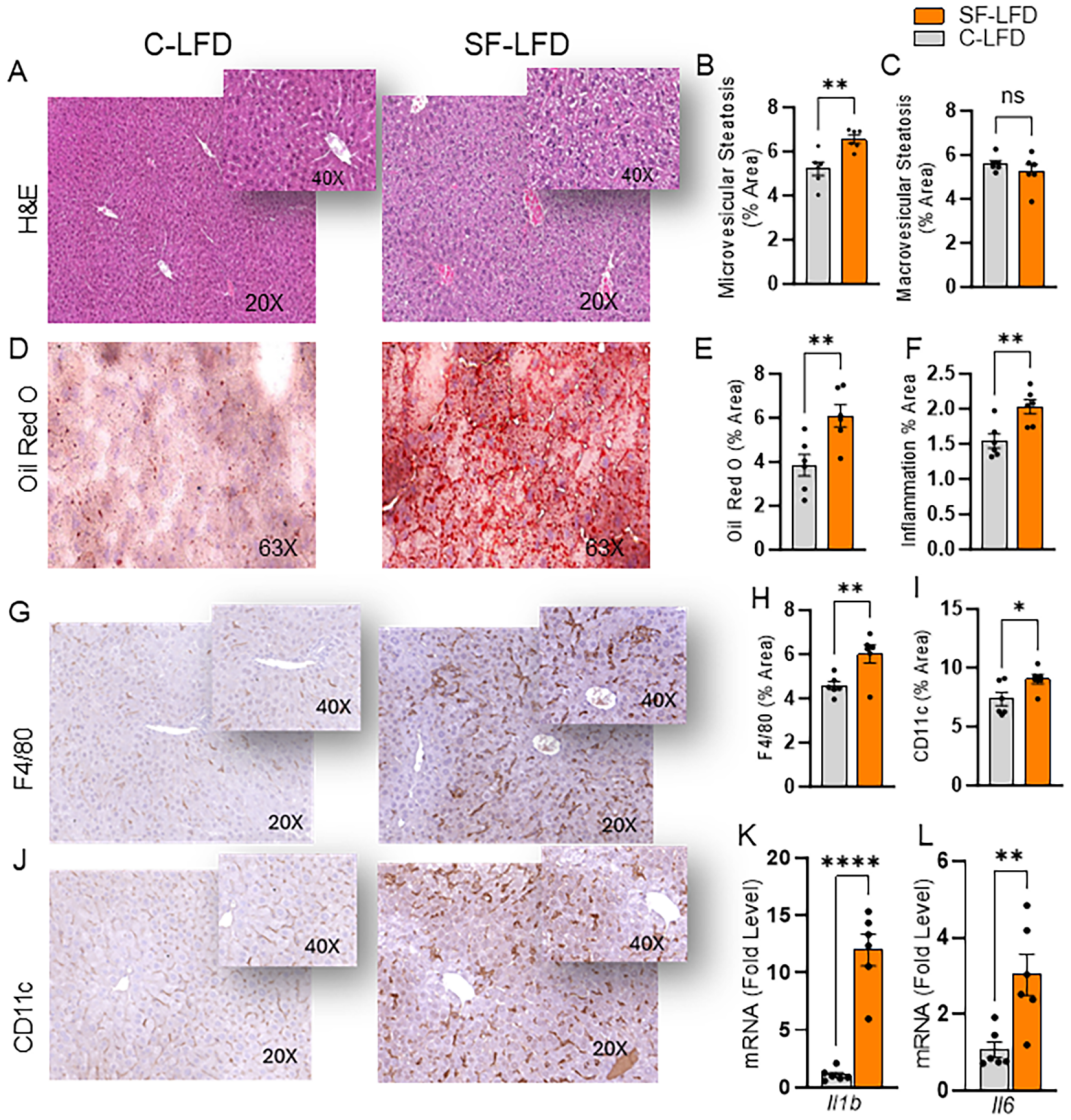
SF-LFD induces liver steatosis and inflammation in mice. Eight- to ten-week-old male C57BL/6 mice (n = 6 per group) were fed a sucrose-free low-fat diet (SF-LFD) or a control low-fat diet (C-LFD) for 16 weeks. **(A)** Representative hematoxylin and eosin (H&E)–stained liver sections (20× magnification). **(B–C)** Quantification of microvesicular and macrovesicular steatosis in C-LFD– and SF-LFD–fed mice. **(D)** Representative Oil Red O staining of liver sections showing lipid accumulation **(E)** Percentage area of Oil Red O staining. **(F)** Quantification of lobular inflammation between dietary groups. **(G)** Representative F4/80 immunostaining of liver sections. **(H)** Percentage of area stained with F4/80. **(J)** Representative CD11c immunostaining of liver sections. **(I)** Percentage area of CD11c staining. **(K–L)** Hepatic mRNA expression of proinflammatory genes (*Il1b* and *Il6*). Data are presented as mean ± SEM. Statistical significance was determined by an unpaired two-tailed Student’s *t-*test: *P *< 0.05*, **P < 0.01, ***P < 0.001, ***P<0.0001, ns, not significant.

Given the well-established gut–liver axis, intestinal inflammation can drive hepatic immune activation and steatosis through translocation of microbial products and systemic inflammatory signaling (28). We therefore hypothesized that SF-LFD–induced colonic inflammation may contribute to hepatic metabolic dysfunction. To test this, we examined the liver phenotype of mice fed SF-LFD or C-LFD. H&E-stained liver sections from SF-LFD–fed mice showed marked accumulation of fat vacuoles and prominent lipid droplets, consistent with hepatic steatosis (**Figure 6A**). In contrast, livers from C-LFD–fed mice exhibited normal histological architecture with minimal lipid deposition. Quantitative scoring demonstrated a significant increase in microvesicular steatosis in SF-LFD mice (**Figure 6B**), whereas macrovesicular steatosis did not differ significantly between groups (**Figure 6C**). Oil Red O staining further confirmed increased hepatic lipid accumulation in SF-LFD–fed mice (**Figures 6D, E**). Additionally, lobular inflammation was significantly elevated in SF-LFD livers compared with controls (**Figure 6F**). To evaluate inflammatory cell infiltration, we stained liver tissue for macrophage marker F4/80, which revealed substantially higher macrophage abundance in SF-LFD livers (**Figures 6G, H**). Similarly, CD11c^+^ inflammatory myeloid cells were more abundant in SF-LFD–fed mice relative to C-LFD controls (**Figures 6I, J**). Consistent with these findings, hepatic expression of the proinflammatory cytokines IL-1β and IL-6 was significantly increased in SF-LFD mice (**Figures 6K, L**).

Together, these findings indicate that sucrose elimination from a low-fat diet induces pronounced hepatic metabolic and immunological alterations, including microvesicular steatosis, macrophage recruitment, and elevated proinflammatory signaling.

## 4 Discussion

In this study, we demonstrated that removal of sucrose from a standard low-fat diet profoundly disrupts metabolic homeostasis and gut–liver immune crosstalk. Contrary to the prevailing assumption that sucrose elimination within a low-fat dietary framework is inherently beneficial, SF-LFD feeding induced insulin resistance, microbial dysbiosis, colonic inflammation, and hepatic steatosis. Likewise, food intake did not differ significantly between the two groups throughout the study, indicating that the observed metabolic differences were independent of caloric intake. While these findings reveal an unexpected physiological response to dietary sucrose removal, the present study demonstrates associations among these changes rather than a direct mechanistic pathway. Metabolically, SF-LFD-fed mice developed significant glucose intolerance and insulin resistance, accompanied by a trend toward elevated fasting blood glucose levels. Although fasting glucose did not reach statistical significance, impairments in OGTT and ITT responses, together with increasing fasting glucose, resemble early prediabetic states observed in humans - a phase that typically precedes the development of persistent fasting hyperglycemia. These findings indicate that sucrose removal, even in the context of low dietary fat, may be sufficient to compromise systemic glucose regulation.

Our microbiome analyses provide a link between dietary sucrose deprivation and metabolic dysfunction. SF-LFD feeding showed a marked reduction in microbial α-diversity and clear compositional separation from C-LFD-fed mice across multiple β-diversity metrics, underscoring a critical role for dietary sucrose in maintaining microbial ecosystem stability. Taxonomic analysis revealed depletion of Firmicutes and short-chain fatty acid (SCFA) producing genera, including *Lactobacillus* and *Ruminococcus*, alongside enrichment of *Bacteroidota*, *Campylobacterota*, and pro-inflammatory genera such as *Helicobacter* and *Odoribacter*. LEfSe analysis further identified enrichment of taxa implicated in bile acid metabolism, epithelial barrier dysfunction, and inflammatory signaling, including *Odoribacter splanchnicus*, *Alistipes* spp., *Pelotomaculum*, and *Cryptobacteroides* (29). Collectively, these changes suggest that sucrose deprivation reduces the availability of fermentable substrate, favoring microbial communities adapted to nutrient stress or bile-rich environments. It is also important to consider that the observed microbial and metabolic alterations may not be attributable exclusively to sucrose removal, as the SF-LFD and C-LFD diets differed in corn starch content to maintain isocaloric conditions. Zhang et al., in their recent review article, highlighted that gut microbial responses to added sugars are strongly influenced by the overall dietary carbohydrate environment, including sugar dose, food form, background diet composition, and replacement macronutrients. Notably, they discussed that several studies replacing starch with simple sugars under isocaloric conditions demonstrated shifts in starch-utilizing microbial taxa, suggesting that changes in starch availability itself can influence microbial ecology (30). Therefore, the microbial dysbiosis and metabolic phenotype observed in the present study may reflect the combined effects of sucrose deprivation and compensatory alterations in dietary starch composition, rather than the absence of sucrose alone. These findings further underscore the complexity of sugar–microbiota interactions and the importance of considering overall carbohydrate composition when interpreting dietary intervention studies. The loss of SCFA-producing bacteria likely compromises gut barrier integrity and immunometabolic signaling, thereby promoting mucosal inflammation and impairing insulin sensitivity (31).SCFA specifically sodium butyrate tested in both prevention and supportive treatment of colorectal cancer (32). Previous studies have shown that dietary carbohydrate quality can influence gut microbial composition and metabolic regulation. In particular, artificial sweeteners such as saccharin and sucralose have been reported to alter microbial community structure in both animal and human studies, with some reports linking these changes to impaired glucose control and inflammatory responses (33). These observations support the concept that modifications in dietary carbohydrate sources may affect host metabolism through changes in the intestinal microbial environment. While earlier studies have mainly associated high-sucrose or high-fat diets with metabolic syndrome (22, 34, 35), our findings extend this paradigm by demonstrating that sucrose elimination within a low-fat dietary context can paradoxically disrupt the gut–metabolic axis, highlighting that metabolic health is governed not only by excess nutrients but also by the availability of fermentable substrates that sustain microbiome function.

Consistent with these microbial changes, SF-LFD-fed mice exhibited significant colonic inflammation, characterized by increased inflammatory cell infiltration and elevated expression of inflammatory mediators, despite no differences in body weight or gross colonic architecture compared with C-LFD-fed mice. These findings indicate that intestinal inflammatory changes can occur in the absence of overt systemic abnormalities. Idiopathic relapsing disorders such as Crohn’s disease (CD) and ulcerative colitis (UC) are characterized by chronic intestinal inflammation, and microbiota analyses of CD and UC samples have shown depletion of commensal bacteria, particularly members of the phyla Firmicutes and Bacteroidetes (36). Although altered microbial metabolites, including SCFA, have been implicated in the regulation of mucosal immune tolerance and inflammatory responses in the colon (31), these metabolites were not measured in the present study, and expression of the tight junction markers ZO-1 and occludin did not show clear differences between groups. Therefore, while the histological and inflammatory findings support intestinal immune activation, the current data do not provide direct evidence of impaired epithelial barrier function. While high-fat, sucrose-rich diets are well recognized drivers of intestinal inflammation and metabolic disease (29, 37–40), our findings suggest that sucrose elimination within a low-fat dietary context may also be associated with altered gut immune homeostasis, underscoring the importance of overall nutrient composition in maintaining intestinal health. In line with the reported association between intestinal inflammation, microbial dysbiosis, and liver pathology (41), SF-LFD feeding was accompanied by hepatic steatosis and inflammatory changes. Mice fed the SF-LFD exhibited greater hepatic lipid accumulation and inflammatory cell infiltration, whereas C-LFD-fed mice showed minimal hepatic abnormalities. Previous studies have predominantly attributed steatosis and hepatic inflammation to high-fat, sugar-rich diets, as described by Ishimoto et al. (42). In the present study, however, similar hepatic changes were observed in mice receiving a low-fat diet lacking sucrose, suggesting that dietary sucrose removal may also be associated with liver injury under certain nutritional conditions. The contribution of intestinal inflammation to liver disease has been emphasized previously (43), although the present study does not directly establish that the intestinal microbial or inflammatory changes caused the hepatic phenotype. Therefore, the relationship between colonic inflammation, microbial alterations, and liver pathology observed here should be interpreted as associative rather than causal. From a translational standpoint, these findings challenge the assumption that sugar restriction alone is uniformly beneficial and underscore the importance of preserving gut microbiome functionality when designing dietary interventions. Low-fat diets lacking fermentable carbohydrates may inadvertently promote dysbiosis, intestinal inflammation, and downstream metabolic and hepatic disease. Collectively, our results highlight that metabolic health is governed not solely by reducing dietary fat or sugar, but by maintaining a diet that supports a resilient, SCFA-producing microbiota and intestinal immune homeostasis.

Additional limitations should also be acknowledged. The sample size was modest, and only a single mouse strain was examined, which may limit broader generalization of these findings. Furthermore, the dietary interventions used in this study may not fully reflect the complexity of human dietary patterns, where sucrose restriction is often accompanied by changes in fiber, starch, or alternative sweetener intake. Therefore, caution is warranted when extrapolating these findings to human nutrition.

In conclusion, our results show that a sucrose-free low-fat diet was associated with alterations in glucose homeostasis, gut microbial composition, intestinal inflammation, and liver pathology in mice. These findings challenge the assumption that sucrose removal within a low-fat diet is necessarily metabolically beneficial and highlight the need for further studies to define the mechanisms underlying these effects.

## Data Availability

The original contributions presented in the study are included in the article. Further inquiries can be directed to the corresponding author.
