# Supplementary material for: Sucrose-free low-fat diet induces metabolic dysfunction through gut dysbiosis and colonic inflammation in mice
Source: Front Immunol. 2026 Jul 21;17:1813722. doi: 10.3389/fimmu.2026.1813722 (PMC13433177; doi:10.3389/fimmu.2026.1813722)
Supplement: Supplementary file 1 [file DataSheet1.pdf]

Supplementary Material

Methods

Histological Analysis

Liver tissue samples mounted on slides were also processed for Hematoxylin-Eosin (H&E) staining and for Oil Red O staining for fat content using standard protocols. Immunohistochemistry (IHC) was performed for F4/80 or CD11c antigen staining. Paraffin-embedded liver tissue sections (4 µm thick) were deparaffinized in xylene and rehydrated using descending grades of ethanol (100%, 95%, and 75%) to water. Antigen retrieval was carried out using target retrieval solution (pH 6.0; Dako, Glostrup, Denmark) by pressure cooker boiling for 8 min and cooling for 15 min. After PBS washing, endogenous peroxidase activity was blocked with 3% H2O2 for 30 min and non-specific antibody binding was blocked with 5% nonfat milk (1 h), followed by 1% bovine serum albumin (BSA) solution (1 h). The samples were incubated overnight at room temperature using primary rabbit polyclonal antibody against F4/80 (1:100 dilution, Abcam® ab100790, pH 6.0, (Cambridge, MA, USA). Or CD11c 1:100 dilution, Abcam® ab219799, pH 6.0, (Cambridge, MA, USA). After washing with PBS (0.5% Tween), samples were incubated for 1 h with secondary, horseradish peroxidase (HRP)-conjugated goat anti-rabbit antibody (EnVision Kit, Dako, Glostrup, Denmark) and color was developed using chromogenic 3,3′-diaminobenzidine (DAB) substrate. Samples were washed in running tap water, lightly counterstained with Harris hematoxylin, dehydrated using ascending grades of ethanol (75%, 95%, and 100%), cleared in xylene, and mounted in dibutylphthalate xylene (DPX). For analysis, digital photomicrographs of tissue sections [20×; PanoramicScan II, 3DHistech, Hungary. URL: https://www.3dhistech.com/products-and-software/hardware/pannoramic-digital-slide-scanners/pannoramic-scan-2/ (accessed on 10 August 2021)] were used to quantify the staining in ten different regions and assess the regional heterogeneity in the tissue samples. The regions were outlined

using Aperio ImageScope software [Aperio Vista, CA, USA. URL: https://aperio-imagescope.software.informer.com/9.0/ (accessed on 10 August 2021)]. Aperio-positive pixel count algorithm (version 9) integrated into Imagescope Software was used to quantify the intensity of specific staining in the region. The number of positive pixels was normalized to the number of total pixels (positive and negative) to account for variations in the size of the region sampled. Color and intensity thresholds were set to detect the immuno-staining as positive and the background as negative pixels. Once set, all slides were analyzed using the same parameters. The resultant color markup of the analysis was confirmed for each slide.

Table S1. Detailed composition of experimental diets (SF-LFD and C-LFD)

	SF-LFD		C-LFD	
	D18060402		D21120801	
	gm	Kcal	gm	kcal
**Protein**	19.1	20	19.1	20
**Carbohydrate**	67.9	71	67.9	71
**Fat**	3.8	9	3.8	9
**Total**		100		100
**kcal/gm**	3.83		3.83	
**Ingredient**	Gm	Kcal	Gm	kcal
**Casein**	200	800	200	800
**L-Cysteine**	3	12	3	12
**Corn Starch**	560	2240	495	1980
**Maltodextrin 10**	150	600	150	600
**Sucrose**	0	0	65	260
**Cellulose, BW200**	50	0	50	0
**Soybean Oil**	40.6	365	40.6	365
**Mineral Mix S10026**	10	0	10	0
**DiCalcium Phosphate**	13	0	13	0
**Calcium Carbonate**	5.5	0	5.5	0
**Potassium Citrate**	16.5	0	16.5	0
**Vitamin Mix V10001**	10	40	10	40
**Choline Bitartrate**	2	0	2	0
**Total**	1060.65	4057	1060.65	4057
**Linoleic Acid (gm)**	21		21	
**Total Calories from Linoleic Acid (Kcal%)**	4.7		4.7	
**% Sucrose**	0		7.1	

Table S1: Primer Assay ID's.

**Gene**	**Assay ID**
IL-1β	Mm00434228_m1
IL-6	Mm00446190_m1
Il-12	Mm01288989_m1
IL-23	Mm00518984_m1
CCL2	Mm00441242_m1
TNF-α	Mm00443258_m1
ROR-γ	Mm01261022_m1
TBX21	Mm00450960_m1
FOXO1	Mm00490671_m1
ZO-1	Mm00503867_m1
Occludin	Mm01349279_m1
GAPDH	Mm99999915_g1

Figure S1: Food intake in mice fed C-LFD and SF-LFD throughout the study period

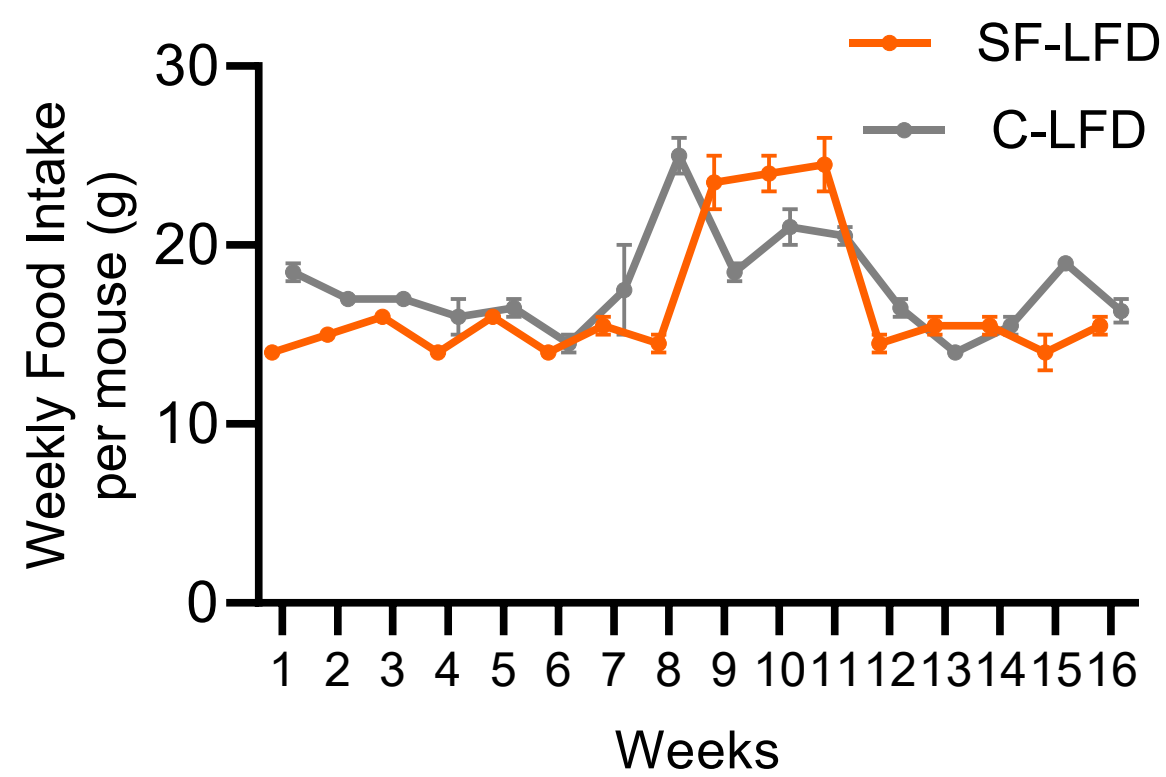

Figure S1. Food intake in mice fed C-LFD and SF-LFD throughout the study period. Food intake was monitored at regular intervals in mice fed either a control low-fat diet containing sucrose (C-LFD) or a sucrose-free low-fat diet (SF-LFD) over the duration of the study. Data are presented as mean ± SEM (n = 6 per group). No significant differences in food intake were observed between the two groups at any time point, indicating comparable caloric consumption.
